# A Computational Approach to Understanding and Predicting the Edulcorant Profile of Glucosyl Steviol Glycosides

**DOI:** 10.3390/foods13121798

**Published:** 2024-06-07

**Authors:** Zhuoyu Zhou, Wei Li, Haijun Wang, Yongmei Xia

**Affiliations:** 1 Key Laboratory of Synthetic and Biological Colloids (Ministry of Education), School of Chemical and Materials Engineering, Jiangnan University, 1800 Lihu Avenue, Wuxi 214122, China; 7200610016@stu.jiangnan.edu.cn (Z.Z.); sanklose@163.com (W.L.); wanghj@jiangnan.edu.cn (H.W.); 2 State Key Laboratory of Food Science and Resources, Jiangnan University, 1800 Lihu Avenue, Wuxi 214122, China

**Keywords:** sensory analysis, sweetness, glucosyl steviol glycoside, steviol glycoside, edulcorant quality, mogroside

## Abstract

Understanding the edulcorant profile of synthetic glucosyl steviol glycosides (GSGs) and rare natural steviol glycosides (SGs) is challenging due to their numerous species and rareness. This study developed a computational model based on the interactions of SG molecules with human sweet and bitter taste receptors (hSTR/hBTR). The models demonstrated a high correlation between the cumulative interaction energies and the perceived sweetness of SGs (R^2^ = 0.97), elucidating the mechanism of the diverse sweetness of SGs. It also revealed that more (within three) glucose residues at the C-13 position of the SG molecule yield stronger sweetness and weaker bitterness. Furthermore, the computational prediction was consistently validated with the known sweetness of GSG and also aligned well with that of several natural mogrosides. Thus, this model possesses a potential to predict the sweetness of SGs, GSGs, and mogrosides, facilitating the application or targeted synthesis of GSGs with desired sensory profiles.

## 1. Introduction

**Figure 1:**
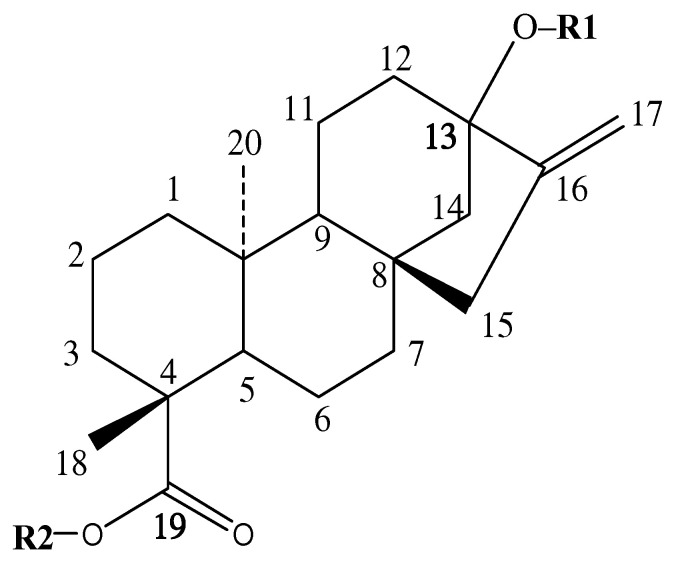
The structure of steviol glycosides.

Steviol glycosides (SGs), a family of tetracyclic diterpenes possessing the same steviol skeleton (Figure 1), have been widely used in the food industry as a calorie-free sweetener or flavor enhancer [1]. To date, at least 64 natural SGs have been identified (FAO JECFA Monographs 20, 2017, ISSN 1817-7077), but only half of them have recorded sweetness data. This is because most of these SGs present in low contents and are difficult to isolate. Structurally, all these steviol glycosides share the same steviol skeleton with diverse glycosyl groups at the C-13 and C-19 positions of the molecule, yielding different sweetness (Table S1).

Nevertheless, well-known SGs such as rebaudioside A (RA), stevioside (St). and rubusoside (Ru) are compromised by their more or less bitter aftertaste [2]. To improve their edulcorant qualities, enzymatic glucosylation of SGs has been developed to produce glucosylated SGs (GSGs) [3,4]. However, sensory analysis, particularly of glycosyl steviol glycosides (GSGs), is complicated due to their complex compositions and similar molecular structures, which make it challenging to separate each component and evaluate their taste profiles [5].

Therefore, understanding or predicting the edulcorant qualities of new or rare SGs and GSGs are crucial, not only because of the sample availability but also for designing new GSGs. In addition to predicting the edulcorant qualities of those SGs and GSGs, what are the mechanisms they performed to make different levels of sweetness and bitterness? Could researchers use an in silico approach to design the structure of a desirable GSG? If a computational approach can be built to predict the edulcorant qualities of GSGs by their structures, it will also help to optimize the synthetic parameter for obtaining GSGs with better edulcorant qualities.

To date, the tertiary structures of some human sweet and bitter taste receptors (hSTR/hBTR) have been elucidated using computational methods [6,7,8,9,10,11], although their functions are not as clear as those of the T1R family in mammals [12]. All these research results attribute the generation of taste to the hydrogen bond interactions between a sweetener molecule and the specific amino acids in the taste receptors [6,7,8,9,10,11,13,14] within different domains [15,16]. For instance, for hSTR, it was reported that glucose can bind to the Venus fly trap domain (VFTD) and amino-terminal domain (ATD) of the T1R2 and T1R3 [15,16]. For hBTR, 25 receptors of the hTAS2R family have been identified as capable of binding with various ligands to produce diverse bitter tastes [17,18]. Consequently, the diverse binding patterns between taste receptors and sweetener molecules (ligands) result in distinctive taste signals, leading to variations in sweetness and bitterness. For example, Acevedo constructed comparative models of human sweet taste receptors to study the interactions with natural sweeteners, including sweet proteins and glycosylated terpenoids [6]. Jaitak et al. utilized docking scores to explain why rebaudioside A is sweeter than stevioside and the bitterness of some SGs and steviol [8,9]. The findings suggest that higher docking scores correlate with increased bitterness; however, no model function was released.

Thus, the impact of glucosyl groups on the edulcorant qualities of SGs/GSGs remains unclear. Clarifying the binding modes between SGs/GSGs and taste receptors will help reveal the relationship between structure and taste in these compounds and identify dominant structures for designing GSGs with favorable taste profiles.

In this study, the impact of glycosyl groups on the sweetness and bitterness of a group of natural SGs (Table S1) and GSGs will be examined through molecular docking with selected sequence domains of human sweet (hT1R2, hT1R3) and bitter (hT2R4) taste receptors. Two molecular docking methods will be compared to establish a correlation between the known sweetness levels of SGs and their interaction energy values. Interaction patterns between SGs/GSGs and the taste receptors will be analyzed to provide insights into the mechanisms influencing their sweetening qualities. Based on the relationship between the known sweetness of SGs and interaction energy values obtained from optimized molecular docking, the edulcorant qualities of GSGs will be discussed and validated. Subsequently, structures of GSGs with improved edulcorant qualities based on the model will be proposed. This research will benefit the application and target production of SGs and GSGs.

## 2. Materials and Methods

### 2.1. Preparation of Ligands

The ligands, including natural steviol glycosides (SGs), glucosyl steviol glycosides (GSGs), and mogrosides were drawn using ChemDraw (ChemDraw 15.0, serial number: 391-475410-8261). The 2D structure of the compound was then prepared for computational analysis using docking with the Prepare Ligands wizard in Discovery Studio (Discovery Studio 2.5.0.9164). To be specific, the CHARMm forcefield was applied, and energy minimization was conducted using the Smart Minimizer algorithm with a maximum of 10,000 steps and an implicit solvent dielectric constant of 80.

### 2.2. Modeling of Sweet Taste Receptors

Amino acid sequences for hT1R2 and hT1R3 were acquired from the NCBI database, with accession numbers NP_689418.2 and NP_689414.1, respectively. Template identification was conducted via a PSI-BLAST search on the Protein Data Bank (PDB), focusing on sequence identity and E-value to prioritize templates closely related to the target sequences. This search highlighted the B chain of the medaka fish taste receptor t1R2a-t1R3 ligand-binding domains (PDB ID: 5X2M) as the most analogous to the hT1R2 and hT1R3 sequences. The closest analogue by sequence, 5X2M, was then chosen as the template to build hT1R models.

The Build Homology Model protocol of Discovery Studio 2.5 was used to construct the models of hT1R2/3. The sequences of target receptors in FASTA format were imported into the Align Multiple Sequences protocol. With the template sequence, initial target (hT1R2/3)-template sequence alignment was generated, and then prediction on the secondary structure was performed according to the alignment. Each modeling process would generate five primary model structures sorted by the probability density function (PDF) of total energy. A model with the lowest PDF of total energy possesses the highest reliability. The initial model was then subjected to loop and side-chain optimization. The restrained minimization of models was performed with the Prepare Protein protocol using the CHARMm force field. Finally, refined models were evaluated with the Ramachandran plot and Verify 3D score [19], and then applied for molecular docking.

### 2.3. Modeling of Bitter Taste Receptors

As for hT2R4, the I-TASSER (Iterative Threading ASSEmbly Refinement) web server was employed for modeling [20]. The modeling process involved extracting consecutive segments from the chosen templates and reassembling them into a full-length model using Monte Carlo simulations [21]. The assembled model was then refined to achieve a low free-energy state and to remove spatial collisions through the application of TM-align [22]. Further refinement was performed to enhance the model’s atomic details, particularly optimizing the hydrogen bonding networks [23]. The final model’s quality was assessed using the TM-score from I-TASSER, and its structural reliability was verified by examining the Ramachandran plot.

### 2.4. Molecular Docking

The models of taste receptors, fully prepared and restrain minimized, were screened for possible binding sites using the Define Site from the Receptor Cavities tool of Discovery Studio. The active sphere was divided by the predicted binding sites to delimit the range where docking happened. Illustrative diagrams of the binding centers for the three receptor proteins are shown in Figure S1. The radii of active spheres of hT1R2, hT1R3, and hT2R4 are 19.5 Å, 20.0 Å, and 20.2 Å, respectively. For comparative study, docking was carried out on the active sphere in Discovery Studio by the CDOCKER and Libdock semi-flexible docking methods, respectively. The specific docking parameters of CDOCKER are as follows: Top Hits (100); Pose Cluster Radius (0.5); Random Conformation (10); Orientations to Refine (10); Maximum Bad Orientations (800); Orientation vdW Energy Threshold (300); Simulated Annealing (true); Heating Steps (2000); Heating Target Temperature (700); Cooling Steps (5000); Cooling Target Temperature (300); and Forcefield (CHARMm). The specific docking parameters of Libdock are as follows: Number of Hotspots (100); Docking Tolerance (0.25); Docking Preferences (High Quality); and Conformation Method (FAST). During the semi-flexible docking, the receptor conformation was fixed and the conformation of the SG ligand could be changed within the allowable range. Accuracy and efficiency of the calculation will be considered simultaneously [24,25]. Finally, the interaction energy (IE) of CDOCKER and the dock score (DS) of the Libdock between the receptor and ligand were calculated. The interaction energy in the docking process is negative, thus CDOCKER takes the absolute values of interaction energies to facilitate comparing them. Higher IE or DS value favors more stable binding between the receptor and ligand.

## 3. Results and Discussion

### 3.1. Template Optimization for the hSTR and hBTR Models

For the hSTR models, specifically the hT1R2/3 models, the selection of the modeling template was refined through a BLAST search, emphasizing sequence identity and E-value in relation to the target sequence. The findings, as outlined in Table 1, underscore that a higher sequence identity and a lower E-value signify a more suitable template for the target sequence. According to Table 1, among the three hSTR models reported with templates 3LMK [8], 2E4U [6], and 1EWK [26], the B chain of 5X2M exhibited the highest sequence identity and notably the lowest E-value, establishing 5X2M_B as the optimal template for constructing the hT1R2/3 models (the validation of models built by other templates is shown in Table S2).

As for the hBTR model, i.e., hT2R4, there are no suitable templates with high sequence identity through BLAST. Consequently, the comparative model for hT2R4 was developed using the I-TASSER web server. I-TASSER selected eight templates (4GRV, 5TJV, 4N6H, 5ZBH, 5G1H, 4DJH, 4BUO, and 5HAS) with sequence identities ranging from 9% to 13%. Despite the suboptimal sequence identities of these templates, the selected template fragments showed a high level of consistency with the target sequence. Thus, I-TASSER facilitated the prediction of the protein fragments’ structure and their alignment into a comprehensive model of the target protein.

### 3.2. Model Building and Assessment

**Figure 2:**
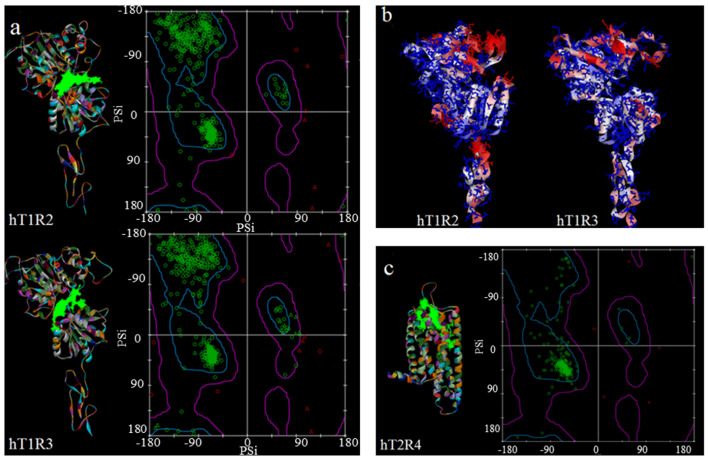
(**a**) The comparative models: Ramachandran plot of hT1R2 and hT1R3; (**b**) The Verify 3D assessment of hT1R2 and hT1R3; (**c**) The comparative models: Ramachandran plot of hT2R4.

The hT1R2 and hT1R3 models were constructed using the Build Homology Model protocol within Discovery Studio 2.5. The structural integrity was scrutinized using Ramachandran plots and Verify 3D scores to ensure accuracy. Analysis via Ramachandran plots (Figure 2a) highlighted that for hT1R2, 96.5% of amino acids resided within the most favored regions and 2.0% in allowed regions, and only 1.5% were classified as outliers, including specific residues such as Gly127, Gly307, Gly429, Gln450, Pro457, Cys486, Gly489, and Gly510. Similarly, for hT1R3, the proportions were 94.5% in the most favored regions, 3.3% in allowed regions, and 2.2% (including Gln10, Gln11, Lys102, Gly126, Gly302, Ala328, Thr360, Gly422, Gly446, Gly489, Arg493, Gly496) as outliers. The distribution closely aligns with findings from prior research, where up to 90% of residues were positioned in the most favored regions [6,8]. In Verify 3D assessment, the models were painted in different colors by their Verify 3D score (Figure 2b). The blue part represents the high score area, and the structure is highly reliable. In contrast, the red part represents the low score area and the structure is unreliable. The Verify 3D score was found to be 0.65 for hT1R2 and 0.95 for hT1R3, indicating a satisfactory level of structural reliability and aligning with historical refinements where models achieved scores ranging from 0.4 to 0.8 [27]. For the hT2R4 model (Figure 2c), the I-TASSER suite was employed to assess its quality, utilizing the TM-Score to gauge the structural resemblance between the constructed model and the natural protein configuration. A TM-Score above 0.5 indicates a model that closely mirrors the natural structure, whereas a score below 0.17 denotes a model with limited similarity [20]. The hT2R4 model achieved a TM-Score of 0.70 ± 0.12, indicating a high level of reliability. Furthermore, the Ramachandran plot revealed that 93.0% of residues fall within the most favored region and 5.3% in the allowed region, while 1.7% (Ser72, Leu121, Pro220, Gly256, Met257) are considered outliers. These findings align with those reported in another study [9], underscoring the accuracy of comparative models. Meanwhile, through comparison with the structures in the AlphaFold 2.0 repository (Table S3), the structure predicted by AlphaFold has a higher Verify 3D score (28%) than the model constructed by I-Tasser (18%), but they are still below 30%, indicating that the model quality has not significantly improved. In addition, when assessing protein non-bonded interactions using ERRAT, the I-Tasser model scored higher than the AlphaFold model (97.25 vs. 95.10). Hence, in this experiment, AlphaFold 2.0 provided a model of similar quality to that of I-Tasser.

In summary, the analysis reveals that over 93.0% of amino acids in the modeled sweet and bitter receptor structures are located within the most favored region, indicating reasonable conformations. On the other hand, fewer than 2.2% of the amino acids fall into the outlier regions, indicating minor conformational discrepancies. These findings affirm the high reliability of the comparative models for further docking studies, which are crucial for exploring potential correlations with sweetness data. This robust validation supports their use in advancing our understanding of receptor–ligand interactions in taste perception.

### 3.3. Molecular Modeling

#### 3.3.1. The Sweetness of Natural SGs and the Interaction with the Taste Receptors

**Figure 3:**
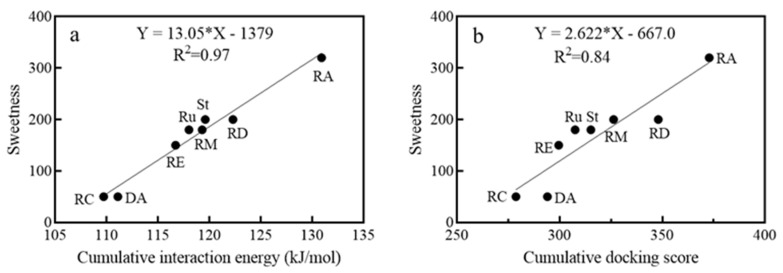
Regression analysis of steviol glycosides’ sweetness and docking results. (**a**): CDOCKER results; (**b**): Libdock results.

To elucidate the sweetness of steviol glycosides (SGs) and their interaction with human sweet and bitter taste receptors (hSTR and hBTR), a computational approach was undertaken. This study involved constructing a model based on existing sweetness data of SGs and examining the relationship between docking results and the sweetening properties. Two docking methods were conducted, namely interaction energy (IE, calculated with CDOCKER) and dock score (DS, calculated with Libdock). The correlation between these metrics and the sweetness levels of SGs was analyzed, as shown in Figure 3, Figure S2 and Table S4.

It was observed that for individual hSTR components (hT1R2 and hT1R3), there was no direct correlation between sweetness and the IE or DS values. Yet, when the IE and DS values from both receptors were combined, a clear correlation with sweetness emerged.

Specifically, Figure 3 highlights that IEs correlate more strongly with the sweetness of steviol glycosides (SGs) (R^2^ = 0.97) than DSs (R^2^ = 0.84), suggesting that IEs are more reliable predictors of sweetness.

Although the availability of bitterness data was constrained, the investigation into bitterness thresholds and relative bitterness revealed a nonlinear correlation [27], where higher interaction energy (IE) or dock score (DS) values were associated with lower bitterness thresholds. This observation aligns with findings from other research that has similarly identified a link between the binding affinity of steviol glycosides (SGs) to bitter taste receptors and their bitterness properties [8,28].

Many researchers aligned the individual IE (T1R to sweetener) or DS to the edulcorant data including sweetness/bitterness of various sweeteners [8,10,13,14] or to the threshold values [8,14], but found hardly any linear relationship. In view of these studies, the hard point was a lack of sweetness/bitterness data. Mayank modeled the interaction between T1R2 and T1R3 with serval SGs [8], using the data reported by Hellfritsch et al. [27], and found that the individual DS (D1, D2) or sum of DS (D1 + D2) was unrelated to the relative bitterness at 1 mM (5–10 folds of the bitterness threshold), but D1 + D2 (assigned as cumulative DS in our experiment) was in a positive proportion to the bitterness threshold. Unfortunately, this finding had not been widely noted. The underlying reason might be that the concentrations chosen to test the sweetness/bitterness data were much higher than the threshold [8], or the structural diversity of the sample sweeteners [8,10,13].

Consequently, the affinity between SGs and bitter receptors can serve as a metric for evaluating the bitterness characteristics.

The analysis also elucidates that maintaining a constant number of C-19 glucosyl moieties while increasing the glucose residue count at C-13 (as observed from Ru to St to RA) leads to elevated cumulative interaction energies (IEs) and diminished IE_hT2R4_ values. Conversely, keeping the glucosyl groups at C-13 unchanged and increasing glucose residues at C-19 (from RA to RD to RM) results in decreased cumulative IEs and IE_hT2R4_. Additionally, among isomers with equal total glucose residues, RA exhibits significantly higher sweetness compared to RE; this is attributed to RA having an additional glucose residue at C-19 and one fewer at C-13. In essence, an increase in glucose residues at C-13 correlates with enhanced sweetness and reduced bitterness in SGs, whereas an increase at C-19 diminishes both sweetness and bitterness.

To further understand how the molecular structure of SGs affects their sweetening properties, we analyzed the interaction patterns between SGs and human sweet and bitter taste receptors, with RA serving as a case study (Figure S3), along with the identification of key amino acid residues involved (Table S5).

The interaction patterns between SGs and taste receptors primarily involve hydrogen bonding (Figure S3), which aligns with previous research [8,9]. Analysis showed that there are 20 amino acid residues in hT1R2 and 17 in hT1R3 involved in these interactions, suggesting that each SG engages with the receptors in unique patterns and poses, potentially leading to different perceptions of sweetness or bitterness (Table S5).

Analysis of the binding sites for hSTR and hBTR reveals significant differences in their spatial configurations, which are crucial for understanding how the structure of SGs influences their taste profiles. Specifically, the hSTR binding site, depicted in Figure S3a as a green area, is located within a wide gap between the ATD domain lobes, providing a spacious environment. This structural arrangement allows ligands to approach and access the binding site from multiple directions with minimal steric hindrance. In contrast, the hBTR binding site, shown in Figure S3c as a green area, is situated between several amino acid helices, offering a much more confined space. As a result, ligands can only access the hBTR binding site from a single direction, encountering significant spatial constraints. This structural variance between hSTR and hBTR binding sites underpins a preference for SG molecules with larger C-13 sides in hSTR, while hBTR exhibits an affinity for SG molecules with smaller C-19 sides. This distinction is pivotal for understanding the impact of SGs’/GSGs’ structure on their sweetening efficiency.

**Figure 4:**
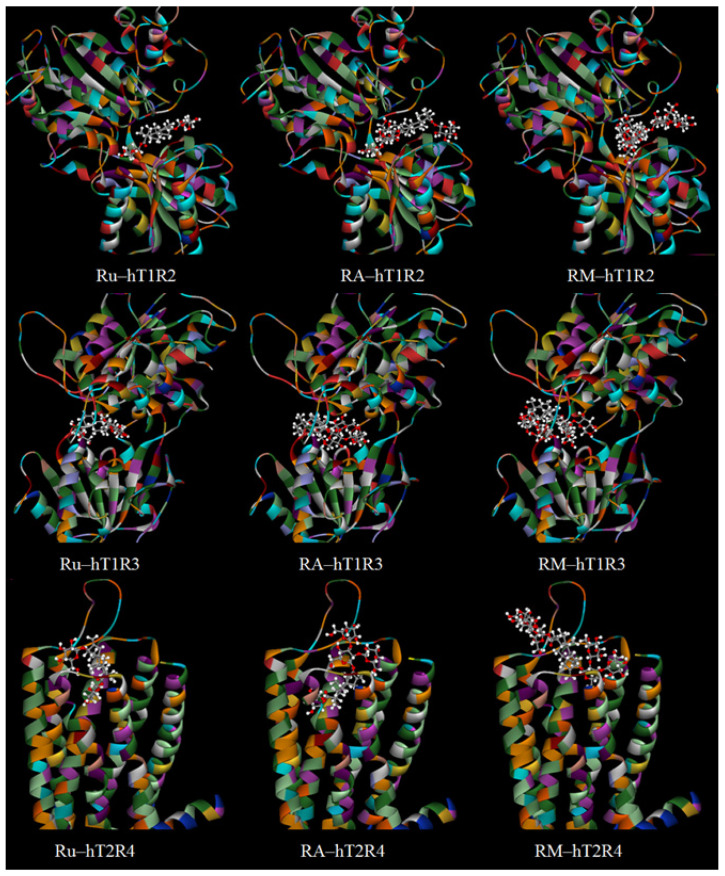
The docking poses of rubusoside (Ru, with one glucose at C13 and one glucose at C19), rebaudioside A (RA, with three glucoses at C13 and one glucose at C19) and rebaudioside M (RM, with three glucoses at C13 and one three glucoses at C19) docked on hT1R2, hT1R3 and hT2R4.

Moreover, the docking poses of Ru, RA, and RM molecules on hSTR and hBTR reinforce the aforementioned observations (Figure 4). For hSTR, SG molecules that possess an abundance of glucose residues at the C-13 position are capable of forming multiple interactions, such as hydrogen bonds with amino acid residues. This results in a higher ligand-receptor interaction energy, which in turn elicits stronger sweetness signals. Therefore, an SG molecule with more glucose residues attached to the C-13 side binds more effectively to hSTR (from Ru to RA). On the other hand, for hBTR, only the glucosyl group at C-19 is small enough to enter its binding site. The presence of additional glucose residues at C-13 not only hinders the formation of strong interactions with amino acid residues but also compromises the stability of the SG-hBTR binding conformation, leading to a reduction in IE and, consequently, diminished bitterness. Increasing the number of glucose residues at C-19 (from RA to RM) does not affect the binding to hSTR but negatively impacts the interaction with hBTR due to spatial constraints, resulting in weaker sweetness and bitterness. This analysis also elucidates why the sweetness of RA, which has more glucose residues at C-13, is significantly stronger than that of RE, which has more glucose residues at C-19. The reduced number of glucose residues at C-19 is more suitable for the tighter binding sites of hBTR, facilitating entry and interaction, thereby influencing the perceived sweetness and bitterness.

#### 3.3.2. The Sweetness Prediction of Glycosylated Steviol Glycosides

To meet the growing demand for improved taste profiles, enormous efforts have been devoted to synthesizing novel glycosyl steviol glycosides. However, the procedure may be costly and time-consuming. In a previous section, the model helped us to understand the differential edulcorant qualities of SGs. Thus, the edulcorant qualities (especially the sweetness) of GSGs (Table 2) might be predictable by calculating their cumulative IE with taste receptors by CDOCKER, of which the GSG with higher cumulative IEs and a lower IE_hT2R4_ would possess stronger sweetness and weaker bitterness.

The molecular structures are graphed in the supporting material as Figure S4.

Table 3 presents the predicted sweetness of GSGs with their cumulative IEs and IE_hT2R4_ calculated by CDOCKER. These GSGs can be obtained from St or RA by enzymatic glucosylation with more glucose residues at C-13 or C-19, and some of them can also be found in nature [2,5,29,30,31,32]. For instance, RI (GSG No. 7 with a calculated sweetness of 224 but no empirical sweetness data reported) and a monoglucosyl rebaudioside A (RA1G, GSG No. 12 with a calculated sweetness of 199) are isomers, and both of them are found in trace amounts in natural *Stevia rebaudiana* leaves making them challenging to separate [33]. According to computational predictions, RA1G exhibits similar cumulative IEs and IE_hT2R4_ values to those of RI (Table 3). Consequently, the taste profile of RA1G is expected to be comparable to that of RI. In practice, the perceived sweetness of enzymatically synthesized RA1G was measured at 200 (prepared from rebaudioside A and sucrose, catalyzed by an alternansucrase from L. citreum CICC23234) [34].

**Figure 5:**
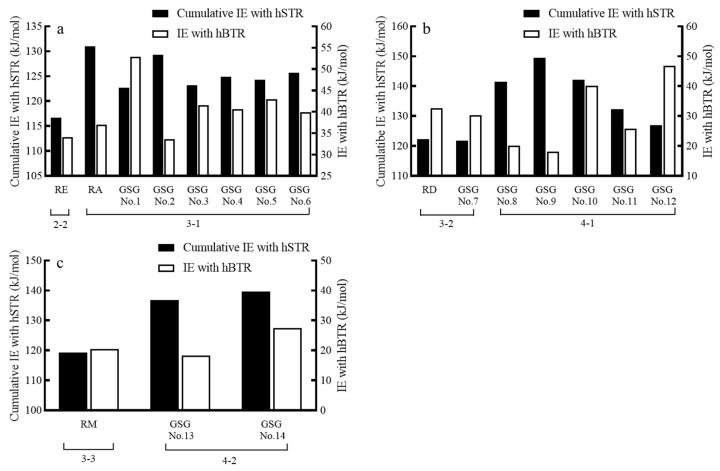
The cumulative IEs and IE_hT2R4_ of glucosyl steviosides. Total glucose residue numbers are (**a**) 4, (**b**) 5, (**c**) 6; in which x-y (such as 2-1, 3-1) represents x glucose residues at C-13 and y glucose residues at C-19.

Therefore, computational modeling can serve as a valuable tool for forecasting and elucidating the sweetness and bitterness of GSG isomers. As shown in Figure 5, for mono-(a), bi-(b), and tri-(c) glucosyl St, the SGs with more glucose residues at C-13 and fewer glucose residues at C-19 showed higher cumulative IEs among the isomers. The outcome may be explained by the interaction dynamics observed between SGs and the hSTR (Figure 4), wherein GSGs with an abundance of C-13 glucose residues readily engage with the receptor’s larger binding sites, fostering enhanced amino acid interactions. Simultaneously, a reduced presence of C-19 glucose residues helps maintain the SG-hSTR binding stability. These factors collectively contribute to heightened cumulative IEs and pronounced sweetness.

Therefore, to enhance the taste profile of GSGs based on St or RA, adding one or two glucose residues at the C-13 position might be an effective approach.

#### 3.3.3. Extending Prediction of Sweetness of Mogrosides

**Figure 6:**
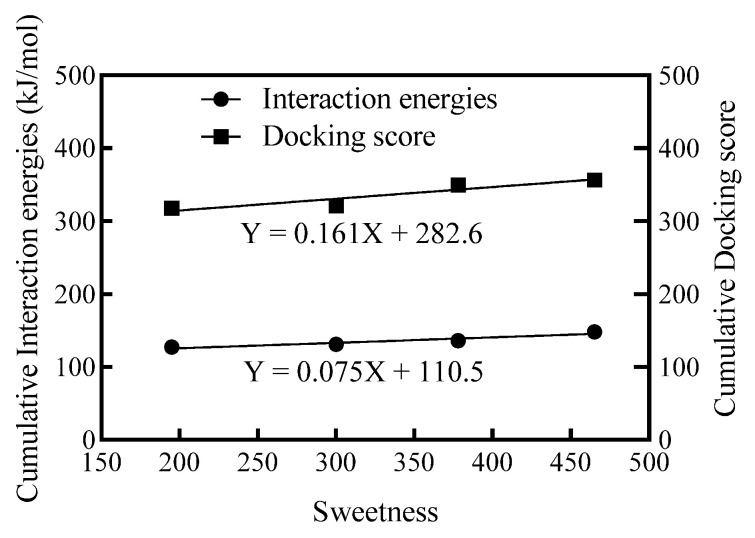
The sweetness of four mogrosides vs. cumulative IEs/cumulative DSs from the hSTRs. For mogrosides, refer to the Supporting Material, Table S6.

In addition to steviol glycosides, numerous other natural sweeteners are being explored for their unique sweetness properties, and investigations into the reasons for their sweetness are also ongoing [35]. Mogrosides are another group of natural sweeteners, and share the same mogrol backbone (Figure S5). Employing similar templates as those used for steviol glycosides (SGs), four mogrosides were docked with hSTRs, and the cumulative IEs and DSs were calculated (Table S6). These values were then correlated with the sweetness data sourced from the literature [36], as depicted in Figure 6. Similarly, both calculated IEs and DSs were highly correlated with sweetness, with an R^2^ of 0.91 (for cumulative IEs) and 0.87 (for cumulative DSs), respectively. Using this predictive model, the sweetness of iso-mogroside V and neomogroside was estimated at 408.83 and 84.6, respectively. These predicted values closely match the taste evaluations reported by Guilin Layn Natural Ingredients Corp., China, which recorded sweetness values of 400 for iso-mogroside V and 100 for neomogroside. However, to ensure the model’s reliability and accuracy across a broader spectrum of mogrosides, further validation with additional compounds is essential.

## 4. Conclusions

To better understand how the molecular structures of steviol glycosides (SGs) and glucosyl steviol glycosides (GSGs) influence their sweetness, and to solve the problem of verifying the sweetness and bitterness attributes of rare SGs and GSGs, a computational method was developed in this study. This approach aimed to correlate the binding affinities of SGs with taste receptors and explain taste variations using an interaction model.

Highly reliable comparative models of human sweet taste (hT1R2, hT1R3) and bitter taste (hT2R4) receptors were constructed using the most suitable templates. Molecular docking between these receptors and natural SGs was performed. The results revealed that the cumulative interaction energies (IEs) and cumulative docking scores (DSs)—the arithmetical sum of the results from hT1R2 and hT1R3—showed a high correlation with perceived sweetness. Regression equations derived from the two docking methods indicated that IEs were more closely related to sweetness perception than DSs.

It was also found that for assigned SGs, more glucose residues at C-19 induce both weaker sweetness and bitterness, while more glucose residues at C-13 generate stronger sweetness and weaker bitterness, which appears to be caused by the different steric hindrance of hSTR and hBTR. Based on the computational predictions, the edulcorant qualities of various GSGs were estimated. Some GSGs may be more desirable as sweeteners compared to rebaudioside A (RA) or their isomers. Adding one or two glucose residues at C-13 on either steviol (St) or RA is predicted to enhance the edulcorant quality of GSGs effectively. This computational approach not only predicts the sweetening properties of GSGs with newly designed structures but also paves the way for the creation and prediction of high-efficiency GSGs, offering promising directions for future sweetener development.

## Figures and Tables

**Table 1:** Screening templates for modeling hT1R2 and hT1R3.

	hT1R2		hT1R3	
	Sequence Identity	E-Value	Sequence Identity	E-Value
5X2M_B	34%	7 × 10^−75^	37%	9 × 10^−101^
3LMK_A	25%	3 × 10^−28^	28%	2 × 10^−22^
2E4U_A	26%	1 × 10^−41^	26%	4 × 10^−43^
1EWK_A	25%	6 × 10^−23^	25%	3 × 10^−24^

**Table 2:** Glucosyl steviol glycosides (GSGs) assigned in this experiment.

GSG No.	GSG Classification	R1 (C-13)	R2 (C-19)
1	/	Glc(α1-3)Glc(β1-2)Glc(β1-	Glc(β1-
2	/	Glc(α1-4)Glc(β1-2)Glc(β1-	Glc(β1-
3	/	Glc(α1-6)Glc(β1-2)Glc(β1-	Glc(β1-
4	/	Glc(β1-2)[Glc(α1-6)]Glc(β1-	Glc(β1-
5	/	Glc(β1-3)Glc(β1-2)Glc(β1-	Glc(β1-
6	RA2	Glc(β1-6)Glc(β1-2)Glc(β1-	Glc(β1-
7	RI	Glc(β1-2)[Glc(β1-3)]Glc(β1-	Glc(β1-3)Glc(β1-
8	/	Glc(β1-2)Glc(β1-2)[Glc(β1-3)]Glc(β1-	Glc(β1-
9	/	Glc(β1-2)Glc(β1-3)[Glc(β1-2)]Glc(β1-	Glc(β1-
10	/	Glc(α1-4)Glc(α1-4)Glc(β1-2)Glc(β1-	Glc(β1-
11	/	Glc(α1-6)Glc(β1-2)[Glc(α1-6)]Glc(β1-	Glc(β1-
12	/	Glc(β1-3)Glc(β1-2)Glc(β1-	Glc(α1-6)Glc(β1-
13	RQ3	Glc(α1-4)Glc(β1-3)[Glc(β1-2)]Glc(β1-	Glc(α1-4)Glc(β1-
14	/	[Glc(α1-4)]_2_Glc(β1-2)Glc(β1-	Glc(α1-4)Glc(β1-

**Table 3:** IE and protein–ligand interactions of hT1R2, hT1R3, and hT2R4 with GSGs.

GSG No. *	IE_hT1R2_ (KJ/mol)	IE_hT1R3_ (KJ/mol)	Cumulative IEs (KJ/mol)	hT1R2-Ligand Interactions	hT1R3-Ligand Interactions	IE_hT2R4_ (KJ/mol)	hT2R4-Ligand Interactions
1	47.32	75.40	122.72	Lys52, Asn130, Thr229	Ser130, Ser153, Glu284, Thr288, Gln372	52.87	Met1, Phe69, Val70, Tyr147, Asn165, Thr166, Thr246
2	64.33	64.95	129.28	Lys52, Asn130, Ser290	Asn51, Asp199, Thr288, Ser289, His371	33.59	Met89, Asp92, Tyr147, Ser185
3	49.60	73.57	123.17	Tyr90, Ser290, Val296, Asn299	His128, Glu131, Gly151, Glu200, Gln309, Gln372	41.56	Asn165, Thr166
4	56.27	68.56	124.83	Lys52, Asn130, Thr229, Glu289, Ser290, Asp294	Ser129, Glu131, Ser153, Thr288	40.66	Met1, Thr166
5	45.59	78.69	124.28	Lys52, Asn130, Ser290, Asp294, Arg370	Ser130, Glu131, Gly151, Asp199, Thr288, His371, Gln372	42.95	Asp92, Ser185
6	50.23	75.43	125.66	Lys52, Glu289, Ser290, Ser367, Arg370	Asn51, Trp55, His128, Ser130, Glu131, Asp199, Asn369	39.91	Asn165, Ser185. Ser243
7	44.87	76.85	121.72	Ser27, Tyr90, Asn130, Ser131, Leu266, Glu289, Ser290, Asp294	Glu28, Gly151, Glu284, Ala285, Gln309, Gln372	30.27	Met89, Tyr147, Thr246
8	58.25	83.24	141.49	Ser27, Asp129, Asn130, Ser131, Ser152, Tyr202, Thr229, Glu289, Arg370	Ser130, Asp199, Glu284, Asp290, Gln309, Asn369, Gln372	20.10	Met89, Asn165, Thr166, Ser185, Ser243, Met259, Ser263
9	66.79	82.76	149.55	Ser27, Asn130, Ser152, Asp265, Glu289, Ser290	Asn51, Glu284, Ala285, Asp290	18.06	Phe69, Met89, Ser263
10	61.31	80.88	142.19	Ala30, Tyr90, Asn130, Asp294, Arg370	His128, Ser130, Ser153, His261, Glu284, Ala285, Thr288	40.05	Met89, Tyr147, Thr246, Met259
11	56.92	75.41	132.33	Lys52, Asp129, Asp200, Thr229, Glu289, Ser290, Arg370	Asn51, Glu131, Glu284, Gln309, His371, Gln372	25.71	Asp92, Tyr147, Asn165, Ser185, Ser263
12	45.46	81.55	127.01	Ser27, Asp129, Asn130, Glu132	Glu131, Gly151, Glu284, Gln309	46.91	Ala9, Glu158, Met259, Ser263
13	60.29	76.58	136.87	Ser27, Lys52, Asn130, Leu266, Glu289, Ser290, Ser445	Asn51, Gly151, Ser153, Thr288, Asn369	18.21	Phe69, Thr246, Ser263
14	52.54	87.06	139.60	Lys52, Thr229, Glu289, Ser290, Ser367, Arg370	Asp199, Thr288, Asp290, Asn369, Leu451	27.41	Ser154, Thr166, Tyr250, Ser263

* The structures of GSGs are listed in Table 2. The absolute values of IE were applied for convenient analysis.

## Data Availability

The original contributions presented in the study are included in the article/supplementary material, further inquiries can be directed to the corresponding author.

## Supplementary Materials

The following supporting information can be downloaded at: https://www.mdpi.com/article/10.3390/foods13121798/s1, Figure S1: the active spheres of (a) hT1R2, (b) hT1R3, (c) hT2R4 models; Figure S2: the cumulative IE, cumulative DS of the steviol glycosides; Figure S3: the interaction patterns of rebaudioside A with hT1R2, hT1R3 and hT2R4; Figure S4: the structure of glycosyl steviol glycoside. Figure S5: structure of the assigned mogrosides; Table S1: the sweetness and structure of common natural steviol glycosides; Table S2: the validation of comparative models of hT1R2 and hT1R3 built by single template; Table S3: comparison of the taste receptor structures modeled by homology and predicted with AlphaFold 2.0; Table S4: Interaction Energy (IE) and Dock Score (DS) of hT1R2, hT1R3 and hT2R4 with natural SGs; Table S5: the protein–ligand interactions of hT1R2, hT1R3 and hT2R4 with natural SGs; Table S6: Interaction Energy (IE) and Dock Score (DS) of hT1R2, hT1R3 and hT2R4 with natural mogrosides. References [37,38,39,40,41] are cited in the Supplementary Materials.

## References

[1] Orellana-Paucar A.M. (2023). Steviol Glycosides from *Stevia rebaudiana*: An Updated Overview of Their Sweetening Activity, Pharmacological Properties, and Safety Aspects. Molecules.

[2] Gerwig G.J., Te Poele E.M., Dijkhuizen L., Kamerling J.P. (2016). Stevia glycosides: Chemical and enzymatic modifications of their carbohydrate moieties to improve the sweet-tasting quality. Adv. Carbohydr. Chem. Biochem..

[3] Zhang R., Tang R., Wang W., Bi J., Xu X., Fan Q., Li Y., Chen Q. (2023). Engineering of cyclodextrin glycosyltransferase improves the conversion efficiency of rebaudioside A to glucosylated steviol glycosides and increases the content of short-chain glycosylated steviol glycoside. Microb. Cell Factories.

[4] Yang L., Ping Q., Yuan Z., Jiang J., Guo B., Liu C., Rao Y., Shi J., Zhang Y. (2023). Highly efficient synthesis of mono-β-1,6-Glucosylated Rebaudioside A derivative catalyzed by glycosyltransferase YjiC. Carbohydr. Res..

[5] Li S., Li W., Xiao Q.Y., Xia Y. (2013). Transglycosylation of stevioside to improve the edulcorant quality by lower substitution using cornstarch hydrolyzate and CGTase. Food Chem..

[6] Acevedo W., Ramirez-Sarmiento C.A., Agosin E. (2018). Identifying the interactions between natural, non-caloric sweeteners and the human sweet receptor by molecular docking. Food Chem..

[7] Singh N., Pydi S.P., Upadhyaya J., Chelikani P. (2011). Structural Basis of Activation of Bitter Taste Receptor T2R1 and Comparison with Class A G-protein-coupled Receptors (GPCRs). J. Biol. Chem..

[8] Mayank, Jaitak V. (2015). Interaction model of steviol glycosides from *Stevia rebaudiana* (Bertoni) with sweet taste receptors: A computational approach. Phytochemistry.

[9] Singla R., Jaitak V. (2016). Synthesis of rebaudioside A from stevioside and their interaction model with hTAS2R4 bitter taste receptor. Phytochemistry.

[10] Zhu Z., Zhang W., Li Z., Zhao W., Liu C., Zhu B., He P., Tang S., Wu Y., Yang J. (2024). Rethinking Sweetener Discovering: Multiparameter Modeling of Molecular Docking Results between the T1R2-T1R3 Receptor and Compounds with Different Tastes. J. Agric. Food Chem..

[11] Zhao S., Ma S., Zhang Y., Gao M., Luo Z., Cai S. (2024). Combining molecular docking and molecular dynamics simulation to discover four novel umami peptides from tuna skeletal myosin with sensory evaluation validation. Food Chem..

[12] Nelson G., Hoon M.A., Chandrashekar J., Zhang Y., Ryba N.J., Zuker C.S. (2001). Mammalian sweet taste receptors. Cell.

[13] Yuan Y., Yiasmin M.N., Tristanto N.A., Chen Y., Liu Y., Guan S., Wang Z., Hua X. (2024). Computational simulations on the taste mechanism of steviol glycosides based on their interactions with receptor proteins. Int. J. Biol. Macromol..

[14] Pich J., Chuquichambi E.G., Blay N.T., Corradi G.B., Munar E. (2020). Sweet and bitter near-threshold solutions activate cross-modal correspondence between taste and shapes of cups. Food Qual. Prefer..

[15] Nie Y., Vigues S., Hobbs J.R., Conn G.L., Munger S.D. (2005). Distinct contributions of T1R2 and T1R3 taste receptor subunits to the detection of sweet stimuli. Curr. Biol..

[16] Lemon C.H. (2024). A non-singularity in sweet taste. Chem. Senses.

[17] Shaji C.S., Saraswathy R. (2023). Taste receptors influencing effective modalities in human health—A cutting edge update on TAS1R and TAS2R receptor polymorphisms in taste perception and disease risk. Nutr. Health.

[18] Naciri L.C., Mastinu M., Crnjar R., Barbarossa I.T., Melis M. (2023). Automated identification of the genetic variants of TAS2R38 bitter taste receptor with supervised learning. Comput. Struct. Biotechnol. J..

[19] Lovell S.C., Davis I.W., Arendall W.B., De Bakker P.I., Word J.M., Prisant M.G., Richardson J.S., Richardson D.C. (2003). Structure validation by ca geometry: Φ, ψ and Cβ deviation. Proteins Struct. Funct. Bioinform..

[20] Roy A., Kucukural A., Zhang Y. (2010). I-TASSER: A unified platform for automated protein structure and function prediction. Nat. Protoc..

[21] Zhang Y. (2008). I-TASSER server for protein 3D structure prediction. BMC Bioinform..

[22] Zhang Y., Skolnick J. (2007). Scoring function for automated assessment of protein structure template quality. Proteins Struct. Funct. Bioinform..

[23] Li Y., Zhang Y. (2009). REMO: A new protocol to refine full atomic protein models from C-alpha traces by optimizing hydrogen-bonding networks. Proteins Struct. Funct. Bioinform..

[24] Gagnon J.K., Law S.M., Brooks C.L. (2016). Flexible Cdocker: Development and application of a pseudo-explicit structure-based docking method within charmm. J. Comput. Chem..

[25] Wu G.S., Robertson D.H., Brooks C.L., Vieth M. (2010). Detailed analysis of grid-based molecular docking: A case study of CDOCKER-A CHARMm-based MD docking algorithm. J. Comput. Chem..

[26] Liu B., Ha M., Meng X.Y., Kaur T., Khaleduzzaman M., Zhang Z., Jiang P., Li X., Cui M. (2011). Molecular Mechanism of Species-dependent Sweet Taste toward Artificial Sweeteners. J. Neurosci..

[27] Hellfritsch C., Brockhoff A., Stähler F., Meyerhof W., Hofmann T. (2012). Human Psychometric and Taste Receptor Responses to Steviol Glycosides. J. Agric. Food Chem..

[28] Bhardwaj V., Singh R., Singh P., Purohit R., Kumar S. (2020). Elimination of bitter-off taste of stevioside through structure modification and computational interventions. J. Theor. Biol..

[29] Kusama S., Kusakabe I., Nakamura Y., Eda S., Murakami K. (2014). Transglucosylation into stevioside by the enzyme system from *Streptomyces* sp.. J. Agric. Chem. Soc. Jpn..

[30] Zhang Y.D., Li W., Lu T., Xia Y.M. (2015). The effect of microwave irradiation on transglycosylation pathway of stevioside with starches or cyclodextrins catalyzed by a cyclodextrin glucanotransferase. J. Mol. Catal. B Enzym..

[31] Guo Q., Zhang T., Wang N., Xia Y., Zhou Z., Wang J.R., Mei X. (2019). RQ3, A Natural Rebaudioside D Isomer, Was Obtained from Glucosylation of Rebaudioside A Catalyzed by the CGTase Toruzyme 3.0 L. J. Agric. Food Chem..

[32] te Poele E.M., Devlamynck T., Jäger M., Gerwig G.J., Van de Walle D., Dewettinck K., Hirsch A.K.H., Kamerling J.P., Soetaert W., Dijkhuizen L. (2018). Glucansucrase (mutant) enzymes from *Lactobacillus reuteri* 180 efficiently transglucosylate Stevia component rebaudioside A, resulting in a superior taste. Sci. Rep..

[33] Geuns J.M.C. (2013). Stevia and Steviol Glycosides.

[34] Wang W. (2020). Synthesis of Monoglucosyl Rebaudioside A and Its Application. Master’s Thesis.

[35] Nakagita T., Matsuya T., Narukawa M., Kobayashi T., Hirokawa T., Misaka T. (2023). Modeling the structure of the transmembrane domain of T1R3, a subunit of the sweet taste receptor, with neohesperidin dihydrochalcone using molecular dynamics simulation. Biosci. Biotechnol. Biochem..

[36] Murata Y., Yoshikawa S., Suzuki Y.A., Sugiura M., Inui H., Nakano Y. (2006). Sweetness characteristics of the triterpene glycosides in *Siraitia grosvenori*. J. Jpn. Soc. Food Sci..

[37] Tao R., Cho S. (2020). Consumer-Based Sensory Characterization of Steviol Glycosides (Rebaudioside A, D, and M). Foods.

[38] Prakash I., Markosyan A., Bunders C. (2014). Development of Next Generation Stevia Sweetener: Rebaudioside M. Foods.

[39] Kinghorn A.D. (2001). Stevia: The Genus Stevia.

[40] Geuns J.M. Stevia and Steviol Glycosides. *Euprint, Heverlee* 2010. https://www.euprint.be/nl/boeken/wetenschappelijke-boeken/stevia-and-steviol-glycosides.

[41] Chatsudthipong V., Muanprasat C. (2009). Stevioside and related compounds: Therapeutic benefits beyond sweetness. Pharmacol. Therap..

