# Supplementary material for: A Computational Approach to Understanding and Predicting the Edulcorant Profile of Glucosyl Steviol Glycosides
Source: Foods. 2024 Jun 7;13(12):1798. doi: 10.3390/foods13121798 (PMC11202765; doi:10.3390/foods13121798)
Supplement: Supplementary file 1 [file foods-13-01798-s001.zip › foods-2996319-supplementary.pdf]

Supporting material

A computational approach to understand and predict the edulcorant profile of glucosyl steviol glycosides

Caption List:

Table S1. The sweetness and structure of common natural steviol glycosides
Table S2. The validation of comparative models of hT1R2 and hT1R3 built by single template
Table S3. Comparison of the taste receptor structures modeled by homology and predicted with AlphaFold 2.0
Table S4. Interaction Energy (IE) and Dock Score (DS) of hT1R2, hT1R3 and hT2R4 with natural SGs
Table S5. The protein–ligand interactions of hT1R2, hT1R3 and hT2R4 with natural SGs
Table S6. Interaction Energy (IE) and Dock Score (DS) of hT1R2, hT1R3 and hT2R4 with natural mogrosides

Figure S1. The active spheres of (a) hT1R2, (b) hT1R3, (c) hT2R4 models
Figure S2. The cumulative IE vs. cumulative DS of the steviol glycosides.
Figure S3. The interaction patterns of rebaudioside A with hT1R2, hT1R3 and hT2R4.
Figure S4. The structure of glycosyl steviol glycoside.
Figure S5. Structure of the assigned mogrosides.

Table S1. The sweetness and structure of common natural steviol glycosides

natural SG	sweetness*	R1 (C-13)	R2 (C-19)
Rebaudioside A	320	Glc(β1-2)[Glc(β1-3)]Glc(β1-	Glc(β1-
Stevioside	200	Glc(β1-2)Glc(β1-	Glc(β1-
Rebaudioside D	200	Glc(β1-2)[Glc(β1-3)]Glc(β1-	Glc(β1-2)Glc(β1-
Rebaudioside M	180	Glc(β1-2)[Glc(β1-3)]Glc(β1-	Glc(β1-2)[Glc(β1-3)]Glc(β1-
Rubusoside	180	Glc(β1-	Glc(β1-
Rebaudioside E	150	Glc(β1-2)Glc(β1-	Glc(β1-2)Glc(β1-
Dulcoside A	50	Rha(α1-2)Glc(β1-	Glc(β1-
Rebaudioside C	50	Rha(α1-2)[Glc(β1-3)]Glc(β1-	Glc(β1-

Table S2. The validation of comparative models of hT1R2 and hT1R3 built by single template

	hT1R2		hT1R3	
	Verify 3D score	Unfavorable residues	Verify 3D score	Unfavorable residues
5X2M	47.8%	2.9%	88.7%	3.0%
3LMK	19.5%	3.7%	14.8%	5.1%
2E4U	15.1%	4.3%	45.4%	3.4%
1EWK	23.2%	2.5%	28.0%	3.7%

Table S3. Comparison of the Taste Receptor Structures Modeled by Homology and Predicted by AlphaFold

	hT1R2		hT1R3		hT2R4	
	Comparative models	AlphaFold models	Comparative models	AlphaFold models	Comparative models	AlphaFold models
Verify 3D score	47.8%	0%	88.7%	56.6%	18.1%	28.4%

	hT2R4	
	Model in this study	AlphaFold model
ERRAT	97.25	95.1

Table S4. Interaction Energy (IE) and Dock Score (DS) of hT1R2, hT1R3 and hT2R4 with natural SGs

Natural SG	Sweetness*	MRL **	IE_{hT1R2} (KJ/mol)	IE_{hT1R3} (KJ/mol)	cumulative IEs (KJ/mol)	DS_{hT1R2}	DS_{hT1R3}	cumulative DSs	IE_{hT2R4} (KJ/mol)	DS_{hT2R4}	Bitterness Threshold (μmol/L) **	Relative bitterness at 1mM**
Rebaudioside A	320	3.7	54.85	76.14	130.99	184.65	188.23	372.88	37.00	139.46	194	1.3
Rebaudioside D	200	4.8	45.71	76.56	122.27	169.19	178.83	348.02	32.70	135.77	162	0.6
Stevioside	200	2.7	60.86	58.78	119.64	161.93	164.37	326.30	45.45	145.05	112	1.4
Rebaudioside M	180	NA	44.75	74.53	119.28	138.40	176.76	315.16	20.46	124.14	NA	NA
Rubusoside	180	1.8	67.16	50.80	117.96	154.67	152.85	307.52	47.32	149.71	61	2.7
Rebaudioside E	150	NA	42.26	74.44	116.70	132.52	166.96	299.48	34.05	140.11	NA	NA
Rebaudioside C	50	1.5	55.08	54.63	109.71	122.95	155.77	278.72	48.38	159.54	49	1.4
Dulcoside A	50	1.1	61.11	49.97	111.08	139.07	154.90	293.97	44.60	163.57	49	1.7

* Data of RM was from the literatures[1,2], and the others were taken from other literatures[3-5].

**MRL, Maximum relative sweetness; Hellfritsch et al., 2012[6].

Table S5. The protein–ligand interactions of hT1R2, hT1R3 and hT2R4 with natural SGs

Natural SG	hT1R2-ligand interactions	hT1R3-ligand interactions	hT2R4-ligand interactions
Rebaudioside A	Ser27, Tyr90, Asp129, Asn130, Ser131, Glu289, Ser290	His128, Gly151, Glu284, Thr288, Gln309	Asn165, Met259, Ser263
Rebaudioside D	Tyr49, Asp129, Ser152, Ser290, Asp294	His128, Glu131, Ser289, Asp290, Asn369, His371	Val85, Thr246
Stevioside	Lys52, Asp129, Asn130, Ser152, Arg365	Glu131, Asp290	Asn165
Rebaudioside M	Lys47, Asn130, Ser198, Thr229, Asp265, Leu266, Thr267, Asn299	Glu200, Val260, His261, Glu284, Ala285	Thr66, Phe168, Ser263
Rubusoside	Ser131, Ser152, Ser290	Val260, Glu284, Gln309	Met89, Thr246
Rebaudioside E	Tyr90, Asn130, Ser131, Asp200	His128, Glu131, Ser153, Glu284, Asp290, Gln372	Arg3, Tyr147, Ser263
Dulcoside A	Tyr90, Asp200, Thr229, Ser290	Ser153, Asp290, Asn369	Tyr250, Ser263
Rebaudioside C	Tyr90, Asp129, Asn130, Asp200, Glu289, Ser290	Glu28, Gly151, Gln309, Gln372	Thr166, Thr246

Table S6. Interaction Energy (IE) and Dock Score (DS) of hT1R2, hT1R3 and hT2R4 with natural mogrosides

Natural mogroside	Sweetness *	IEhT1R2 (KJ/mol)	IEhT1R3 (KJ/mol)	Cumulative IEs (KJ/mol)	DShT1R2	DShT1R3	cumulative DSs	IEhT2R4 (KJ/mol)	DShT2R4
Mogroside Ⅲ	195	54.29	72.96	127.25	106.73	210.84	317.57	53.59	194.23
Mogroside Ⅳ	300	49.66	81.48	131.14	121.37	199.84	321.21	44.98	185.90
Mogroside Ⅴ	378	51.47	84.57	136.04	139.10	210.88	349.98	44.32	213.55
Siamenoside Ⅰ	465	55.93	92.17	148.10	145.79	210.65	356.44	69.13	203.12

* Sweetness of mogrosides was reported in the literature[7].

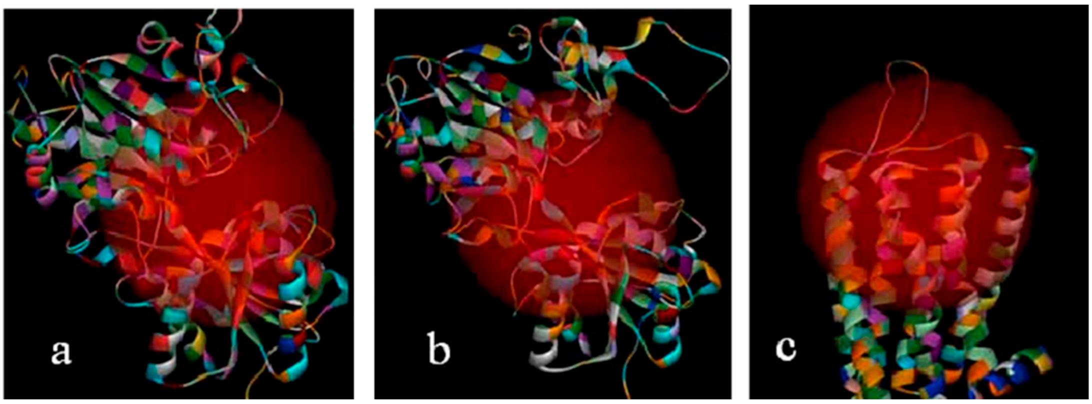

Figure S1. The active spheres of (a) hT1R2, (b) hT1R3, (c) hT2R4 models

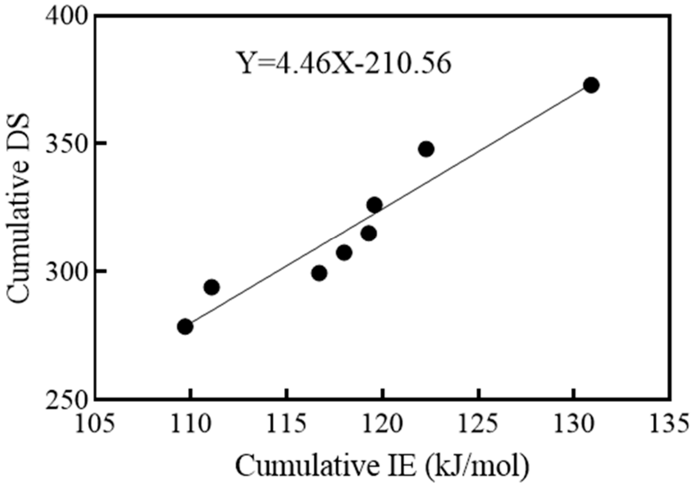

Figure S2. The cumulative IE vs. cumulative DS of the steviol glycosides.

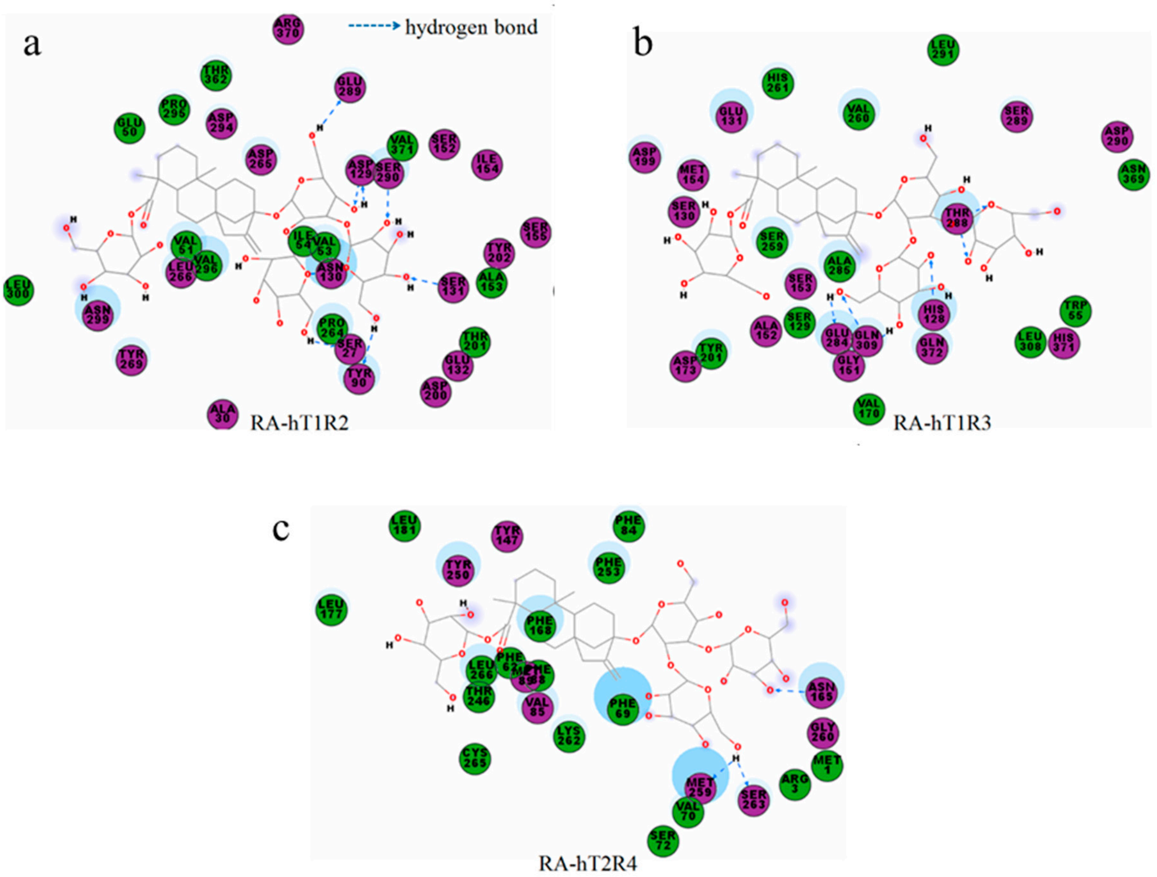

Figure S3. The interaction patterns of rebaudioside A with hT1R2, hT1R3 and hT2R4.

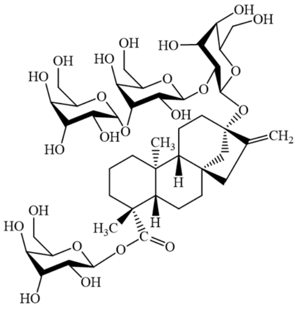

GSG No.1

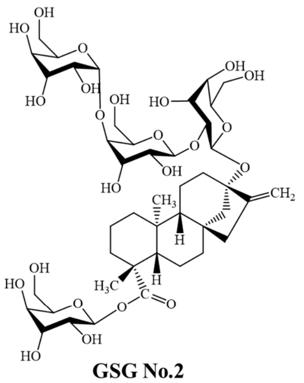

GSG No.2

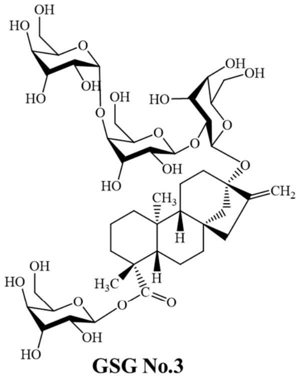

GSG No.3

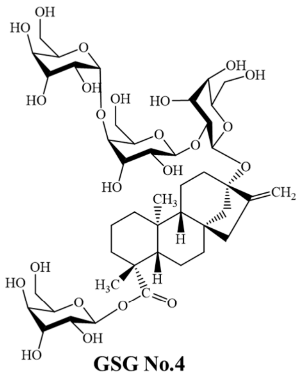

GSG No.4

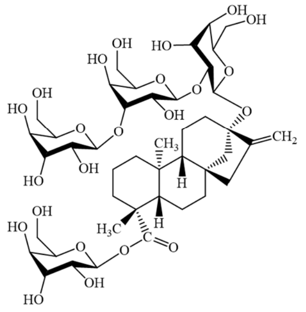

GSG No.5

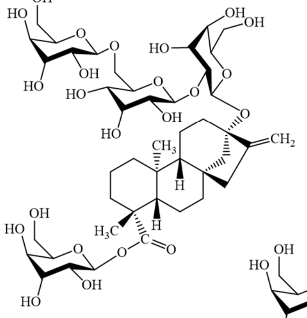

GSG No.6

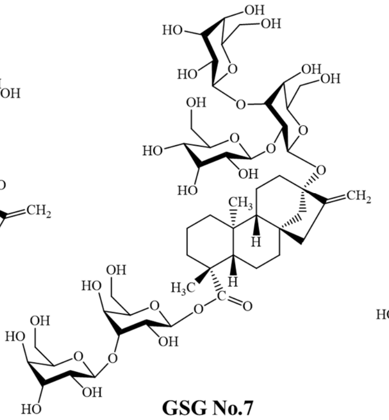

GSG No.7

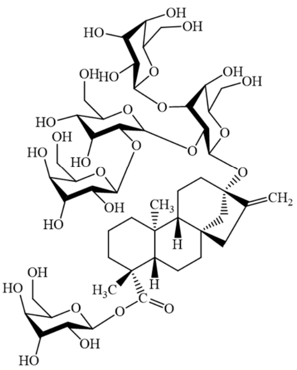

GSG No.8

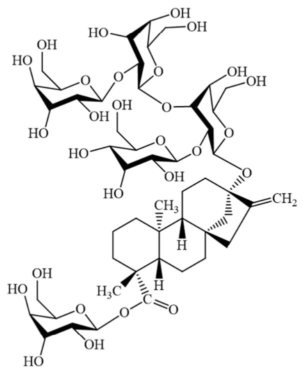

GSG No.9

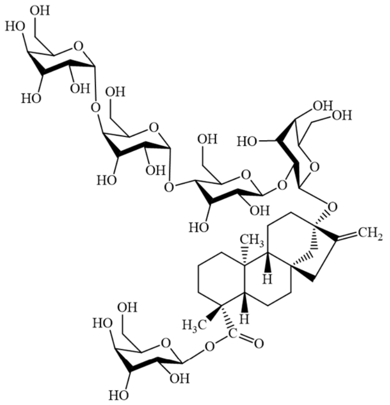

GSG No.10

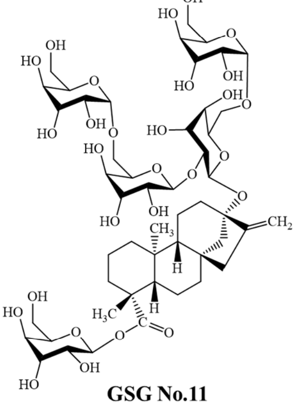

GSG No.11

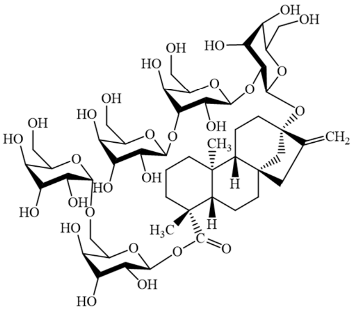

GSG No.12

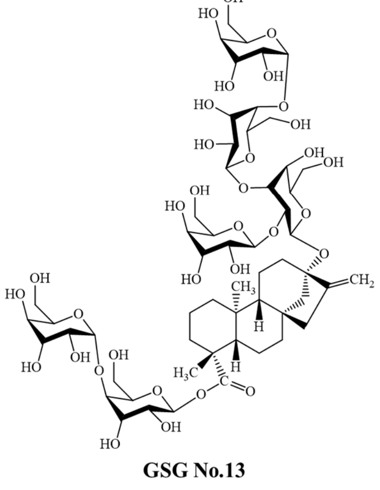

GSG No.13

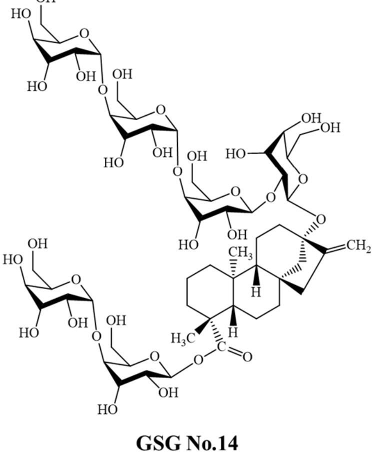

GSG No.14

Figure S4. The structure of glycosyl steviol glycoside.

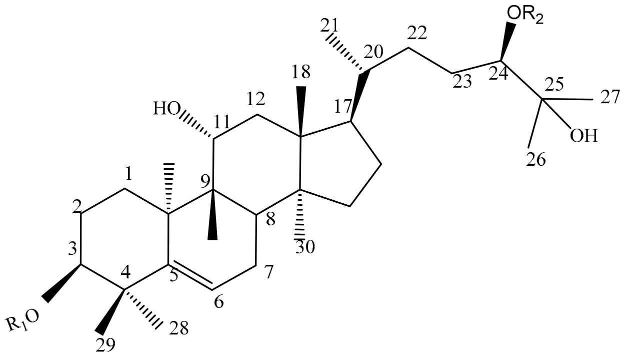

Mogroside	R1 (C-3)	R2 (C-24)
Mogroside III	Glc(β1-	Glc(β1-6)Glc(β1-
Mogroside IV	Glc(β1-6)Glc(β1-	Glc(β1-2)Glc(β1-
Mogroside V	Glc(β1-6)Glc(β1-	Glc(β1-2)[Glc(β1-6)]Glc(β1-
SiamenosideI	Glc(β1-	Glc(β1-2)[Glc(β1-6)]Glc(β1-

Figure S5. Structure of the assigned mogrosides

Reference

1. Tao, R.; Cho, S. Consumer-Based Sensory Characterization of Steviol Glycosides (Rebaudioside A, D, and M). *Foods* **2020**, *9*, doi:10.3390/foods9081026.
2. Prakash, I.; Markosyan, A.; Bunders, C. Development of Next Generation Stevia Sweetener: Rebaudioside M. *Foods (Basel, Switzerland)* **2014**, *3*, 162-175, doi:10.3390/foods3010162.
3. Kinghorn, A.D. *Stevia: the genus Stevia*; CRC Press: 2001.
4. Geuns, J.M. Stevia and steviol glycosides. *Euprint, Heverlee* **2010**.
5. Chatsudthipong, V.; Muanprasat, C. Stevioside and related compounds: Therapeutic benefits beyond sweetness. *Pharmacology & Therapeutics* **2009**, *121*, 41-54, doi:10.1016/j.pharmthera.2008.09.007.
6. Hellfritsch, C.; Brockhoff, A.; Stähler, F.; Meyerhof, W.; Hofmann, T. Human Psychometric and Taste Receptor Responses to Steviol Glycosides. *Journal of Agricultural & Food Chemistry* **2012**, *60*, 6782-6793.
7. Murata, Y.; Yoshikawa, S.; Suzuki, Y.A.; Sugiura, M.; Inui, H.; Nakano, Y. Sweetness characteristics of the triterpene glycosides in Siraitia grosvenori. *J Jpn Soc Food Sci* **2006**, *53*, 527-533.
